# Clinically Deployable Handwriting Biomarkers of Parkinson's Disease via Multiscale Attention and Bayesian–Genetic Optimization

**DOI:** 10.1002/brb3.71457

**Published:** 2026-04-28

**Authors:** Khosro Rezaee, Ali Khalili Fakhrabadi

**Affiliations:** ^1^ Department of Biomedical Engineering Meybod University Meybod Iran; ^2^ Department of Electrical Engineering, Kerman Branch Islamic Azad University Kerman Iran

**Keywords:** attention networks, deep learning, explainable AI, handwriting analysis, Parkinson's disease

## Abstract

**Introduction:**

Subtle PD motor abnormalities can be underappreciated in examination but are overtly present in handwriting. Spiral, meander, and wave drawings are noninvasive, low‐cost methods for capturing PD motor signatures and are easily collected on a tablet. Deep learning is a natural framework to decode such features, but most handwriting‐based PD classifiers have not been thoroughly optimized or tested for generalization, leaving their clinical utility unverified.

**Methods:**

PD‐MGMA‐DSCNN is a multiscale gated multi‐head attention depthwise separable convolutional network operating on a unified RGB image of spiral, meander, and wave tasks. A PD‐specific Bayesian–genetic optimization scheme (PD‐BayGA) was used to tune a suite of key architectural and training hyperparameters. Model decisions were interpreted using SHAP‐based superpixel attribution maps to link discriminative regions to previously described Parkinsonian handwriting abnormalities.

**Results:**

On the PaHaW dataset, PD‐MGMA‐DSCNN achieved 98.23% accuracy on the held‐out internal test set, outperforming CNN, RNN, 3D CNN, and ensemble baselines reported in the literature. Using the finalized configuration developed on PaHaW, external evaluation on the independent HandPD dataset yielded 99.42% accuracy, together with high ROC–AUC and precision–recall performance under clinically motivated rule‐out and rule‐in thresholds. These findings support the potential use of a three‐zone risk stratification scheme within a standardized image‐based drawing framework.

**Conclusions:**

PD‐MGMA‐DSCNN provides state‐of‐the‐art performance in handwriting‐based PD detection while remaining a compact, systematically optimized model with clinically interpretable explanations. The framework may support tablet‐based PD screening or triage in movement‐disorder clinics and longitudinal monitoring, and serves as a practical starting point for incorporating digital handwriting biomarkers into clinical PD care.

## Graphical abstract

Tablet‐based handwriting tasks (spiral, meander, and wave) are transformed into unified images and analyzed using PD‐MGMA‐DSCNN, a lightweight multiscale gated attention network. Bayesian–genetic optimization improves performance, while SHAP attribution maps provide interpretable handwriting biomarkers for Parkinson's disease screening.

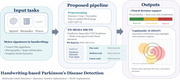

## 1 Introduction

Parkinson's disease (PD) is a chronic, progressive neurodegenerative movement disorder characterized by bradykinesia, rigidity, tremor, and impaired handwriting. These symptoms progressively reduce quality of life and complicate early screening and diagnosis in routine clinical settings. Because handwriting abnormalities can be captured noninvasively and at low cost, handwriting and drawing tasks have attracted growing interest as digital biomarkers for PD screening and monitoring (Zhao et al. 2023; Białek et al. 2024; Aldhyani et al. 2024). Recent deep learning studies using offline images or online handwriting trajectories have reported strong diagnostic performance, indicating that handwriting contains discriminative Parkinsonian motor signatures (Huang et al. 2024; Pragadeeswaran and Kannimuthu 2024). At the same time, translational studies in digital neurology have shown that moving from promising research results to practical screening tools requires not only high accuracy, but also robustness, interpretability, and computational efficiency (Tolosa et al. 2026).

Despite recent progress, three limitations remain common in handwriting‐based PD detection. First, many existing deep models are tailored to specific datasets, task types, or acquisition settings, and their performance may degrade under different protocols, centers, or subject cohorts. Even sophisticated task‐specific or multimodal architectures can remain sensitive to dataset bias, augmentation strategy, or hardware variability, raising concerns about generalizability beyond the training distribution (Zhao et al. 2023; Białek et al. 2024; Huang et al. 2024; Pragadeeswaran and Kannimuthu 2024; Tolosa et al. 2026; Zhu et al. 2025). Second, although many high‐performing models have been reported, patient‐level explanations that clearly relate model decisions to clinically meaningful handwriting abnormalities—such as tremor‐related jaggedness, micrographia, or spiral deformation—are still limited (Tolosa et al. 2026). This contrasts with broader PD research in imaging, voice, and clinical variables, where explainable AI tools such as SHAP, LIME, and related attribution methods are increasingly used to improve interpretability (Tabashum et al. 2024; Rabie and Akhloufi 2025; Esan et al. 2025; Jin et al. 2025; Egbo et al. 2025; Aladhadh 2025; Chaddad et al. 2023). Third, although principled hyperparameter optimization is known to be important for stable and reproducible CNN performance across datasets and training protocols (Raiaan et al. 2024), many handwriting‐based PD models still rely on manual trial‐and‐error or simple search strategies.

These limitations—restricted cross‐dataset generalization, insufficient interpretability, and ad hoc hyperparameter tuning—continue to hinder the practical development of handwriting‐based PD screening systems. Motivated by this gap, we aim to develop a framework that generalizes more reliably across datasets and task conditions, provides clinically interpretable visual explanations, and incorporates principled hyperparameter optimization within the learning pipeline. In this study, we focus on image‐based analysis of drawing tasks that are shared or harmonizable across the PaHaW and HandPD datasets. Specifically, the final framework operates on standardized 256 × 256 × 3 image representations derived from spiral, meander, and wave‐type inputs, enabling a unified processing pipeline for samples originating from different acquisition formats. We therefore do not claim to model the full range of online handwriting kinematics, but rather to provide a robust and interpretable image‐based approach for clinically relevant drawing patterns.

To address these aims, we propose PD‐MGMA‐DSCNN (Parkinson's detection via multiscale gated multi‐head attention depthwise separable CNN), a depthwise–attention fusion architecture coupled with PD‐specific Bayesian–genetic (PD‐BayGA) hyperparameter optimization and SHAP‐based interpretability. The methodological novelty of the proposed framework lies not in the use of attention alone, but in the integration of four complementary design choices: a lightweight depthwise separable convolutional backbone for computational efficiency, a three‐branch multiscale gated multi‐head attention (MGMA) module for adaptive fusion of fine and coarse handwriting features, a PD‐BayGA optimization strategy for systematic tuning of architecture and training parameters, and SHAP‐based superpixel attribution maps for visual interpretation of model decisions. This combination is designed to jointly capture local stroke irregularities and global shape distortions while remaining compact enough for deployment‐oriented settings.

The main contributions of this study are fourfold. First, we introduce a compact multiscale attention‐based architecture for handwriting‐based PD detection. Second, we integrate PD‐BayGA to optimize key architectural and training hyperparameters under different augmentation and tuning regimes. Third, we evaluate the proposed framework across subject‐independent splits, multiple training settings, and cross‐dataset experiments on PaHaW and HandPD. Fourth, we provide SHAP‐based visual explanations that link model predictions to clinically relevant abnormalities in spiral, meander, and wave drawings. Together, these contributions aim to improve the generalizability, interpretability, and practical utility of deep learning–based handwriting biomarkers for PD, while recognizing that broader prospective and multicenter validation remains necessary before routine clinical adoption.

The remainder of this article is organized as follows. Section 2 describes the related work. Section 3 presents the datasets, task representation, preprocessing pipeline, and the proposed PD‐MGMA‐DSCNN framework. Section 4 reports the experimental design and results. Section 5 discusses the findings, interpretability, clinical relevance, comparative performance, and limitations. Section 6 concludes the article.

## 2 Related Work

Handwriting‐based PD detection has been studied using both online dynamic signals and offline images, evolving from classical feature‐engineering pipelines to end‐to‐end deep learning. In online settings, several studies model pen position, pressure, velocity, or acceleration using temporal or frequency‐based representations. Sequence models combining 1D convolutions with recurrent units have shown improved performance over conventional machine‐learning baselines by explicitly capturing handwriting dynamics (Díaz et al. 2021). Other approaches have used dynamic handwriting descriptors or spectral and cepstral representations to characterize PD‐related motor abnormalities, often reporting strong diagnostic results on tablet‐acquired datasets (Kamran et al. 2021; Moustansir et al. 2026; Lu et al. 2025). These methods demonstrate the value of online kinematic information, but they typically depend on high‐quality sensors, nontrivial signal processing, or richer temporal streams that are not always available in practical image‐based screening settings (Díaz et al. 2021; Kamran et al. 2021; Moustansir et al. 2026; Lu et al. 2025).

In parallel, many studies have applied CNN‐based models to offline handwriting images from datasets such as PaHaW and HandPD. These include ensembles of fine‐tuned 2D CNNs (Gazda et al. 2022), hybrid CNN–classifier pipelines (Varalakshmi et al. 2022), attention‐enhanced CNN architectures (Zhao and Li 2023), restricted Boltzmann machine–based classifiers (Thakur et al. 2023), transformer‐style models with coordinate attention (N. Wang et al. 2023), EfficientNet‐based frameworks (Razaq et al. 2025), and lightweight encoder–decoder variants such as improved LinkNet–GhostNet (Pradeep et al. 2025). Collectively, these studies confirm that offline drawings such as spirals and waves contain highly discriminative PD signatures and that deep models can achieve strong accuracy on public benchmarks (Gazda et al. 2022; Varalakshmi et al. 2022; Zhao and Li 2023; Thakur et al. 2023; N. Wang et al. 2023; Razaq et al. 2025; Pradeep et al. 2025). However, several limitations remain recurrent across this literature. Many methods are optimized for a single task, acquisition condition, or dataset; many rely on relatively complex or computationally heavy architectures; and many are evaluated under settings that provide limited evidence of robustness across cohorts, protocols, or deployment scenarios (Gazda et al. 2022; Varalakshmi et al. 2022; Zhao and Li 2023; Thakur et al. 2023; N. Wang et al. 2023; Razaq et al. 2025; Pradeep et al. 2025).

A complementary line of work has investigated multimodal, optimization‐based, and translational frameworks. Attention‐based fusion of handwriting with clinical variables has been explored to improve prediction beyond handwriting‐only models (Benredjem et al. 2025). Other studies have combined handcrafted features with feature selection or metaheuristic optimization strategies, including Firefly and related approaches, to reduce dimensionality and improve classification efficiency (Shyamala and Navamani 2025; Soleimanidoust et al. 2025). At the translational level, systems such as NeuroDiag have demonstrated the feasibility of tablet‐based acquisition and workflow integration for PD support tools (Ngo et al. 2024), while transfer‐learning studies have explored more lightweight classifiers for spiral drawings (Farhah 2024). These efforts are important for moving toward practical deployment, but they still face common challenges, including dependence on specific task types, limited multicenter validation, and incomplete treatment of explainability and generalizability (Ngo et al. 2024; Farhah 2024; Benredjem et al. 2025; Shyamala and Navamani 2025; Soleimanidoust et al. 2025).

Several review papers have reached similar conclusions. Reviews of handwriting and voice‐based PD detection emphasize that digital biomarkers are promising, but also note that datasets are often small, imbalanced, and heterogeneous in protocol and acquisition setting, making fair benchmarking and external validation difficult (Aouraghe et al. 2023; Islam et al. 2024). Broader reviews of artificial intelligence in PD and handwriting analysis likewise highlight recurring concerns around overfitting, bias, explainability, and regulatory readiness (Aouraghe et al. 2023; Islam et al. 2024; Marano et al. 2025). Taken together, the literature suggests that high accuracy alone is insufficient for clinically useful handwriting‐based PD systems; robust evaluation, transparent interpretation, and efficient implementation are also required.

Against this background, our work is positioned at the intersection of four needs that are not fully addressed together in prior studies: compact image‐based modeling, adaptive multiscale feature fusion, principled hyperparameter optimization, and clinically interpretable visual explanation. Unlike prior methods that focus primarily on dynamic online signals, single‐task offline images, or accuracy‐oriented attention mechanisms alone (Díaz et al. 2021; Kamran et al. 2021; Moustansir et al. 2026; Gazda et al. 2022; Varalakshmi et al. 2022; Zhao and Li 2023; Thakur et al. 2023; N. Wang et al. 2023; Ngo et al. 2024; Farhah 2024; Lu et al. 2025; Ravichandran et al. 2026; Razaq et al. 2025; Benredjem et al. 2025; Pradeep et al. 2025; Shyamala and Navamani 2025; Soleimanidoust et al. 2025), the proposed PD‐MGMA‐DSCNN framework combines a predominantly depthwise separable convolutional backbone with a three‐branch MGMA module, a PD‐BayGA strategy, and SHAP‐based superpixel explanation maps. This integrated design is intended not only to improve predictive performance, but also to offer several practical advantages over prior attention‐based CNN models: lower computational cost and model complexity than heavier convolutional or transformer‐style architectures, adaptive scale selection across fine, medium, and coarse handwriting patterns rather than attention at a single feature level, and a unified image‐based pipeline that combines systematic tuning with clinically meaningful visual interpretation. The framework is designed for standardized image representations of spiral, meander, and wave‐type inputs, with the aim of improving cross‐dataset robustness, interpretability, and deployment‐oriented efficiency in handwriting‐based PD detection.

## 3 Proposed Model

To automatically differentiate the handwriting of the individuals with PD and healthy controls, we design a specific deep learning model named PD‐MGMA‐DSCNN. The model is inspired by the requirements that the model should jointly learn the fine‐grained stroke irregularities (e.g., tremor, jagged contours, micro‐strokes) and coarse global abnormalities (e.g., micrographia, spiral deformation, shape instability), and should be lightweight for execution on tablets or digital pens used in clinics and homes. The key component of PD‐MGMA‐DSCNN is a depthwise–attention fusion module, in which a fully depthwise‐separable convolutional backbone extracts multiscale features of handwriting, which are fused by three gated multi‐head attention branches so the network can adaptively attend to the most informative scale for each sample.

### 3.1 Handwriting Dataset and Image‐Based Representation

Two established and complementary handwriting datasets, PaHaW (Drotár et al. 2014) and HandPD (Pereira, Pereira, Silva, et al. 2016), were used in this work. The PaHaW database comprises online recordings of handwriting by 75 subjects, including 37 with PD (18 women, 19 men) and 38 healthy individuals (18 women, 19 men). Each subject carried out eight different tasks, including trigrams, bigrams, unigrams, an Archimedean spiral, a set of Czech cursive words (e.g., “lektorka,” “porovnat,” “nepopadnout”), and a sentence. The recordings of handwriting were collected using a digitizing tablet, which records the *x*‐ and *y*‐coordinates of the pen in on‐surface and in‐air motions. A stroke is defined by a sequence of points from a pen‐down event to a pen‐up event. The HandPD database comprises offline examination images from the Parkinson's Clinic at the Faculty of Medicine of Botucatu, São Paulo State University, Brazil. This dataset contains 736 handwritten images, 368 in the control group (18 healthy subjects, 6 men, 12 women; 2 left‐handed, 16 right‐handed) and 368 in the patient group (74 individuals with PD, 59 men, 15 women; 5 left‐handed, 69 right‐handed). These two complementary datasets, which together provide rich spatial and temporal information of handwritten content, have been widely used in the literature for handwriting‐based PD detection. We used their samples for the development and validation of the proposed approach to help provide a comprehensive view of motion fluency, pressure, and velocity patterns of PD handwriting.

To improve the clinical interpretability of the study, we further clarify the extent of subject‐level clinical information available from the source datasets. For PaHaW, the source publications report neurologist‐confirmed PD and provide cohort‐level clinical descriptors including disease duration, Modified Hoehn and Yahr/UPDRS‐based severity information, and levodopa‐equivalent dose summaries. However, these public sources do not provide a complete harmonized set of subject‐level annotations for all symptom dimensions that would be desirable for model stratification, such as explicit tremor subtype, micrographia status, bradykinesia severity, or detailed medication‐state subgrouping for every sample used in downstream image‐based studies. For HandPD, the publicly available dataset documentation provides demographic and acquisition information, including age, sex, handedness, and the spiral/meander task structure, but does not provide sufficiently detailed subject‐level metadata regarding tremor prevalence, micrographia, bradykinesia severity, or medication ON/OFF state at the time of acquisition. Accordingly, in the present work the PD cohorts should be interpreted as benchmark diagnostic groups defined at the dataset level, rather than as clinically stratified subcohorts with complete symptom‐level phenotyping. This point is important when considering clinical generalization, and it motivates future validation on prospectively collected cohorts with richer neurological annotation.

Although the original PaHaW and HandPD databases contain a broader range of handwriting and drawing materials, the present study did not use all source tasks for classification modeling. Instead, the final image‐based pipeline retained only the subset of drawing inputs that could be harmonized across datasets and represented consistently in the unified RGB format used by PD‐MGMA‐DSCNN. For PaHaW, although the original database includes eight tasks, only the retained spiral‐, meander‐, and wave‐type representations were used in the final subject‐level fused input. For HandPD, only the retained spiral, meander, and wave drawings that satisfied the preprocessing and representation protocol were included in the present analysis. Accordingly, the effective analytic inputs used for model development and evaluation correspond to the retained task subset summarized in Table 1, rather than to the full range of source‐dataset tasks.

**TABLE 1:** Summary of the source datasets, retained task subsets, class distribution, and effective dataset usage in the present study.

Dataset	Original source tasks	Retained task subset used in final pipeline	Group	Original dataset size	Effective inputs included in final analysis	Use in model development and evaluation
PaHaW	Eight handwriting/drawing tasks in the original database	Spiral‐, meander‐, and wave‐type representations harmonized and fused into a single subject‐level RGB input	Healthy	38 subjects	38 subject‐level fused inputs	Primary development dataset (subject‐independent split): Training/validation/hyperparameter optimization + held‐out internal test
	Eight handwriting/drawing tasks in the original database	Spiral‐, meander‐, and wave‐type representations harmonized and fused into a single subject‐level RGB input	PD	37 subjects	37 subject‐level fused inputs	Primary development dataset (subject‐independent split): Training/validation/hyperparameter optimization + held‐out internal test
HandPD	Source database contains 736 images across drawing tasks	Retained spiral, meander, and wave drawings mapped to the standardized image representation	Healthy	18 subjects; 368 control images	368 retained drawing images	Independent external evaluation dataset used only after model development on PaHaW
	Source database contains 736 images across drawing tasks	Retained spiral, meander, and wave drawings mapped to the standardized image representation	PD	74 subjects; 368 PD images	368 retained drawing images	Independent external evaluation dataset used only after model development on PaHaW

Importantly, the final PD‐MGMA‐DSCNN model did not use the full set of source‐dataset handwriting tasks as direct inputs. Rather, the final model operated only on the unified image‐based representations of spiral‐, meander‐, and wave‐type drawings. Thus, cursive‐word, sentence, trigram, bigram, and unigram tasks from the original PaHaW source dataset were not used as direct inputs in the final classification model. The same retained input definition was used consistently for internal model development and external evaluation.

Because both PaHaW and HandPD contain multiple handwriting and drawing materials, we explicitly summarize in Table 1 the subset of tasks retained for the present study, together with the corresponding healthy/PD distributions and the way these data were used in model development and evaluation. This was done to remove ambiguity regarding the relationship between the original source datasets and the final harmonized inputs used by PD‐MGMA‐DSCNN.

Table 1 clarifies that the final experiments were based on the selected drawing inputs retained in the unified image‐based pipeline, rather than on the full range of source tasks. It also makes explicit the distinct roles of the two datasets in the evaluation protocol, thereby improving the transparency and reproducibility of dataset usage in this study. Specifically, PaHaW was used as the primary dataset for model development, including subject‐independent splitting into training, validation, and held‐out test subsets, as well as PD‐BayGA‐based hyperparameter optimization. By contrast, HandPD was used only as an independent external dataset to assess cross‐dataset generalization, rather than for model selection or tuning.

The handwriting samples from PaHaW (online recordings converted to images) and HandPD (offline scanned images) were processed using the same preprocessing pipeline to standardize their characteristics before being used as inputs to the proposed network. The preprocessing stage involved denoising the raw images to remove background artifacts and enhance image contrast for segmentation of the inked regions, followed by image cropping to remove unnecessary margins and scaling and rotation normalization to reduce inter‐subject variability.

**FIGURE 1:**
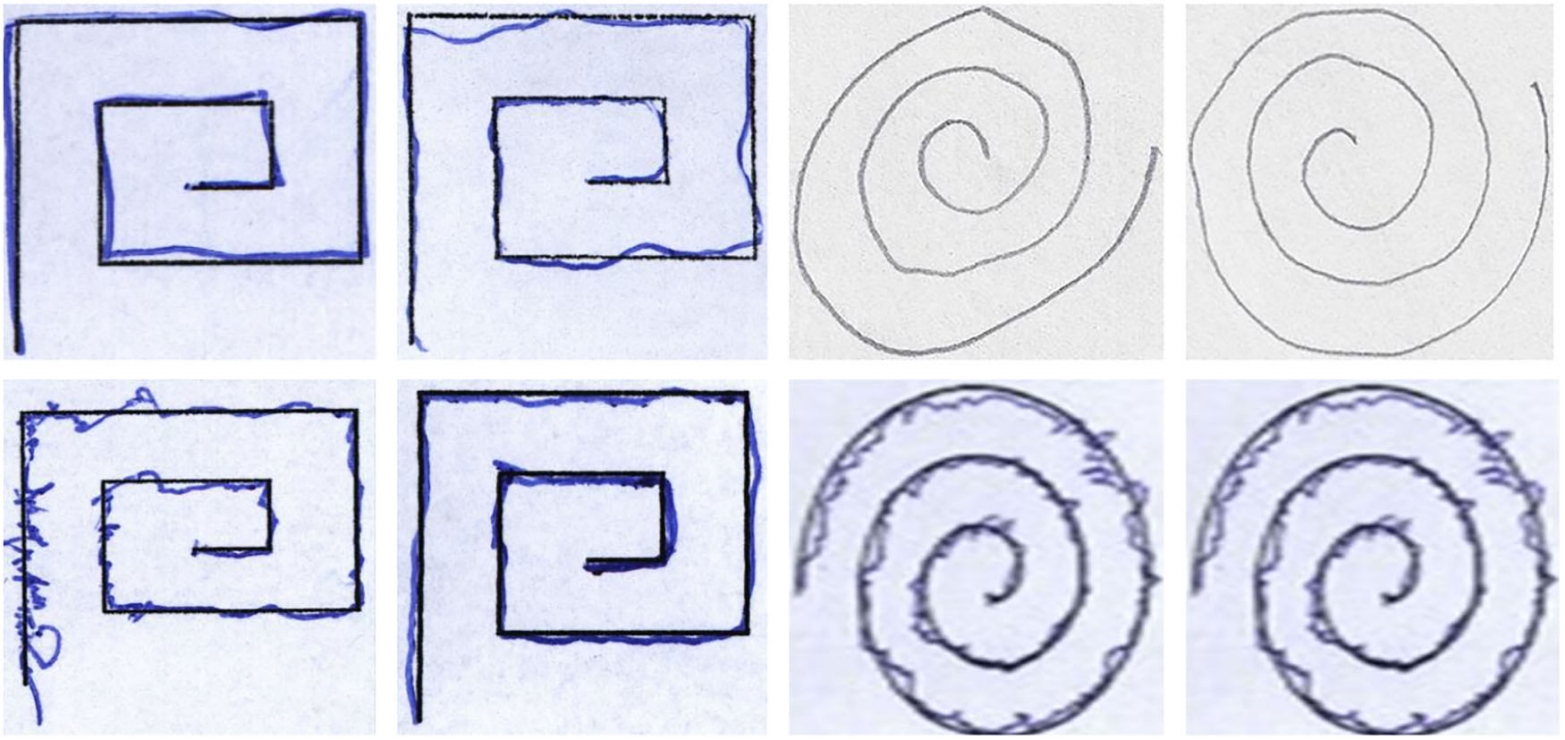
Representative examples of retained drawing inputs used in the unified image‐based pipeline. The illustrated examples show spiral and meander drawings from the HandPD (Pereira, Pereira, Silva, et al. 2016; Pereira, Weber, Hook, et al. 2016; Pereira, Pereira, Papa, et al. 2016) dataset; wave‐type inputs were also included in the final model although not all retained input types are shown in this figure. The first row displays examples from healthy controls, whereas the second row shows drawings from PD cases.

Each example (or, in the case of PaHaW, a fused combination of relevant tasks collected from the same subject) was then rescaled to a fixed spatial resolution and encoded as a 256 × 256 × 3 RGB image, in which the three channels represent complementary visual views of the handwriting content (e.g., different tasks or different filtered versions of the same sample). Several representative examples of these input images are shown in Figure 1. These images constitute the inputs to the PD‐MGMA‐DSCNN framework (Section 3.2).

### 3.2 Proposed PD‐MGMA‐DSCNN Framework

We propose PD‐MGMA‐DSCNN, a lightweight handwriting‐based PD classifier that combines a depthwise separable convolutional backbone with a three‐branch MGMA module. The backbone extracts hierarchical stroke‐ and shape‐level features, while the attention module adaptively fuses fine, medium, and coarse representations for binary PD versus healthy‐control classification. The detailed architecture is described in Sections 3.2.1–3.2.3.

#### 3.2.1 Overall Architecture and Depthwise–Attention Fusion

Let $X^{\left(0\right)}\in R^{H_{0}\;\times \;W_{0}\;\times \;C_{0}}$ denote the input handwriting image, where $H_{0}=W_{0}=256$ and $C_{0}=3$. The proposed PD‐MGMA‐DSCNN framework applies a sequence of five convolutional stages S1,…,S5 to extract hierarchical feature maps, followed by three attention branches, a fusion block, global average pooling, and a final softmax classifier for binary discrimination between PD and healthy controls. Each convolutional stage $S_{\ell }$ implements a mapping:

(1) $$X^{\left(\ell \right)}=f^{\left(\ell \right)}\;\left(X^{\left(\ell -1\right)};\Theta^{\left(\ell \right)}\right),\;\ell =1,\text{…},5,$$

where $\Theta^{\left(\ell \right)}$ denotes the trainable parameters (convolutional kernels and biases) in that stage, and $f^{\left(\ell \right)}\left(\cdot \right)$ is realized predominantly by depthwise separable convolutions followed by batch normalization, nonlinear activation, and max‐pooling. From selected intermediate stages (e.g., 3,S4,S5), we extract three multiscale feature maps:

(2) $$F_{1}\in R^{H_{1}\times W_{1}\times C_{1}},\;F_{2}\in R^{H_{2}\times W_{2}\times C_{2}},\;F_{3}\in R^{H_{3}\times W_{3}\times C_{3}}$$

corresponding to fine, medium, and coarse scales of handwriting representation. Each feature map $F_{b}$ (branch index ∈{1,2,3}) is processed by a branch‐specific projection function $g_{b}\left(\cdot \right)$, implemented by pointwise convolutions and pooling, to obtain a unified resolution and channel depth:

(3) $$Z_{b}=g_{b}\;\left(F_{b};\Phi_{b}\right)\in R^{7\times 7\times d},\;d=192$$

where $\Phi_{b}$ contains the parameters of branch b. This corresponds to the Poi_Conv2D and pooling operations reported for Atn1‐Atn3 in Table A1 (see Appendix A). Each projected feature map $Z_{b}$ is then passed to a gated multi‐head attention operator $A_{b}\left(\cdot \right)$ (detailed in Section 3.2.3), which models long‐range dependencies among spatial locations and modulates the contribution of each scale:

(4) $${\tilde{Z}}_{b}=A_{b}\;\left(Z_{b};\Psi_{b}\right),\;b=1,2,3$$

with $\Psi_{b}$ denoting the attention parameters. The outputs of the three branches are fused along the channel dimension using concatenation, yielding:

(5) $$F_{\mathrm{fused}\;}=\;\mathrm{Concat}\left({\tilde{Z}}_{1},{\tilde{Z}}_{2},{\tilde{Z}}_{3}\right)\in R^{7\times 7\times D},\;D=3d.$$

The fused feature tensor $F_{\mathrm{fused}\;}$ is globally aggregated by a global average pooling operator GAP(·), producing a compact feature vector $z\in R^{D^{\prime }}$ (with $D^{\prime }=32$ in the concrete configuration):

(6) $$z_{c}=\frac{1}{HW}\;\sum_{i=1}^{H}\sum_{j=1}^{W}F_{\mathrm{fused}\;}\left(i,j,c\right),\;c=1,\text{…},D^{\prime }$$

where H=W=7 after fusion and channel reduction. This vector is fed into the final dense layer parameterized by weights $W\in R^{C\times D^{\prime }}$ and biases $b\in R^{C}$ to obtain the logits $\in R^{C}$:

(7) s=Wz+b,C=2

followed by the softmax function to produce the class posterior probabilities

(8) $$p_{k}=\frac{\exp\left(s_{k}\right)}{\sum_{j=1}^{C}\exp\left(s_{j}\right)}\;,\;k=1,\text{…},C$$

where k=1 corresponds to PD and k=2 to healthy. During training, these probabilities are used with the ground‐truth labels in a binary cross‐entropy loss (see Section 3.3) to optimize all parameters

(9) $${\left\{\Theta^{\left(\ell \right)}\right\}}_{\ell =1}^{5},{\left\{\Phi_{b},\Psi_{b}\right\}}_{b=1}^{3},W,b$$

in an end‐to‐end manner. The layer‐wise detailed configuration of the proposed architecture, that is, blocks, layer names, output tensor sizes and parameters, are summarized in Table A1 (see Appendix A). As shown in the table, the backbone S1–S5 mainly consists of depthwise separable convolutions, and only a few standard Conv2D layers in Stages S3–S5, which results in a very compact and computationally efficient feature extractor. The three‐branch gated multi‐head attention module on top of this lightweight backbone operates on the multiscale feature maps to materialize the proposed depthwise‐attention fusion design: multiscale representations from a depthwise‐separable CNN are adaptively fused by attention to jointly exploit local stroke irregularities and global handwriting abnormalities of PD.

#### 3.2.2 Depthwise Separable and Pointwise Convolutions

Let $X\in R^{H\times W\times M}$ denote an input feature map with spatial size H×W and M channels, and let $Y\in R^{H\times W\times N}$ be the output with N channels. A standard 2D convolution layer with kernel size K×K and kernels $K\in R^{K\times K\times M\times N}$ computes:

(10) $$\begin{array}{ccc} & & Y\left(i,j,n\right)=\sum_{u=1}^{K}\;\sum_{v=1}^{K}\sum_{m-1}^{M}K\left(u,v,m,n\right)X\left(i+u-1,j+v-1,m\right),\\ & & \;n=1,\text{…},N \end{array}$$

which requires $K^{2}MN$ parameters and on the order of $HWK^{2}MN$ multiply‐accumulate operations. The pointwise step then performs a 1×1 convolution across channels to mix these intermediate maps U,

(11) $$Y\left(i,j,n\right)=\sum_{m\;-\;1}^{M}\;K_{\mathrm{pw}}\left(m,n\right)U\left(i,j,m\right),\;n=1,\text{…},N$$

with $K_{\mathrm{pw}}\in R^{M\times N}$. This linear combination produces richer feature representations that integrate information from multiple stroke channels, enabling the network to capture more complex handwriting patterns. The total parameter count and computational cost of this factorized layer are proportional to $K^{2}M+MN$, instead of $K^{2}MN$ for a standard convolution. The ratio of operations between depthwise separable and standard convolution can be approximated by computational cost ratio (CCR):

(12) $$\mathrm{CCR}\;=\frac{K^{2}M+MN}{K^{2}MN}=\frac{1}{N}+\frac{1}{K^{2}}$$

which is much smaller than 1 for typical settings (e.g., K=3,N≥64). A more detailed derivation of the depthwise separable formulation and its computational advantage is provided in Appendix B.

#### 3.2.3 MGMA for PD Handwriting

**FIGURE 2:**
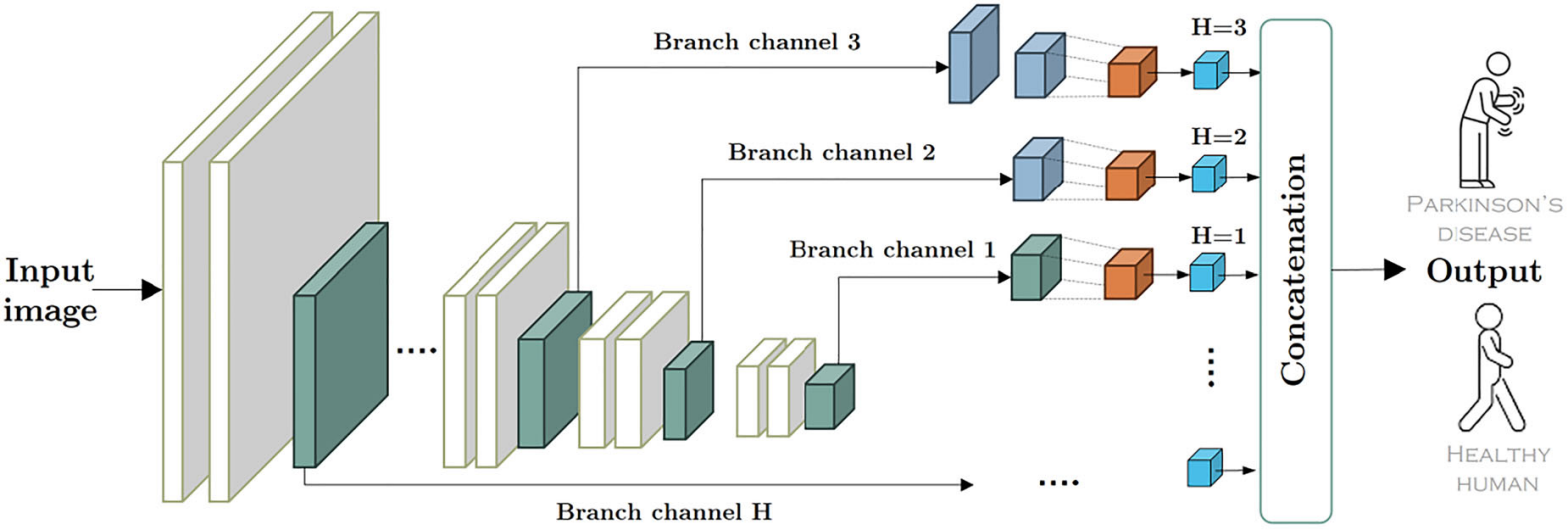
Architecture of the proposed multiscale gated multi‐head attention (MGMA) module, showing branch‐specific projections, gating, and multi‐head attention across fine, medium, and coarse feature scales.

Figure 2 illustrates the proposed three‐branch MGMA module, which operates on feature maps extracted from different depths of the backbone (e.g., Stages S3−S5). As described in Section 3.2.1, these stages provide feature maps $F_{1},F_{2},F_{3}$ at progressively coarser spatial resolutions and higher channel depths, corresponding to fine, medium, and coarse handwriting scales. Each branch channel in Figure 2 receives one of these feature maps and first applies a sequence of pointwise convolutions and, if necessary, pooling operations to project it to a common spatial and channel size,

(13) $$Z_{b}=g_{b}\;\left(F_{b};\Phi_{b}\right)\in R^{7\times 7\times d},\;b\in \left\{1,2,3\right\},d=192$$

where $g_{b}\left(\cdot \right)$ denotes the branch‐specific projection function and $\Phi_{b}$ its parameters. This harmonisation step (shown by the middle blocks in Figure 2) ensures that all branches provide feature maps with identical dimensions before entering the attention mechanism. Each projected feature map $Z_{b}$ is then reshaped into a sequence of L=7×7 tokens of dimension $d_{,\;}$ forming a matrix $X_{b}\in R^{L\times d}$. A multi‐head self‐attention block with H heads (Figure 2, right‐hand colored blocks) is applied to model long‐range dependencies between different spatial regions of the handwriting. To adaptively weigh the contribution of each scale, PD‐MGMA‐DSCNN employs scale‐specific gating before concatenation. For each branch, a global descriptor $u_{b}\in R^{d}$ is obtained by global average pooling over ${\tilde{Z}}_{b}$, and a scalar gate $\alpha_{b}$ is computed via a small shared gating network:

(14) $$u_{b}=\;\mathrm{GAP}\left({\tilde{Z}}_{b}\right),\;\;\alpha_{b}=\frac{\exp\left(w_{g}^{⊤}\sigma \left(W_{g}u_{b}+b_{g}\right)\right)}{\sum_{j-1}^{3}\exp\left(w_{g}^{⊤}\sigma \left(W_{g}u_{j}+b_{g}\right)\right)}\;,\;b=1,2,3$$

where $W_{g},b_{g},w_{g}$ are shared gating parameters and σ(·) is a nonlinear activation. The softly normalized weights $\alpha_{b}$ (indicated by the labels H=1,2,3 in Figure 2) allow the network to emphasize fine‐scale stroke features‐such as tremor or jagged edges‐when they are highly discriminative, or to focus on coarse‐scale shape patterns‐such as micrographia or spiral deformation‐when those cues dominate.

The gated multiscale representation feeding the classifier is then

(15) $$F_{\mathrm{MSA}}=\;\mathrm{Concat}\left(\alpha_{1}{\tilde{Z}}_{1},\alpha_{2}{\tilde{Z}}_{2},\alpha_{3}{\tilde{Z}}_{3}\right)$$

which is subsequently processed by the fusion, global average pooling, and dense layers described in Section 3.2.1. By combining a depthwise‐separable backbone (responsible for efficient multiscale feature extraction) with this gated multi‐head attention module (responsible for adaptive scale selection and long‐range dependency modeling), the proposed design realizes the depthwise‐attention fusion that forms the central innovation of PD‐MGMA‐DSCNN for Parkinson's handwriting analysis. Additional mathematical details of the MGMA mechanism are provided in Appendix C.

### 3.3 Training Procedure and Hyper‐Parameter Settings

For all experiments, the PD‐MGMA‐DSCNN model was trained in a supervised fashion using the binary cross‐entropy loss and the Adam optimizer. The network was initialized with He‐normal initialization and trained from scratch on the PaHaW training data under the subject‐independent development protocol described below. The initial learning rate was set to $1\times 10^{-3}$, with a cosine‐annealing decay schedule and a minimum learning rate of $1\times 10^{-6}$. Mini‐batches of 32 images were used, and the maximum number of epochs was fixed to 150. To prevent overfitting, we employed early stopping based on the validation F1‐score with a patience of 15 epochs. Regularization included $L_{2}$ weight decay of $1\times 10^{-4}$ applied to all convolutional and dense layers, as well as dropout with a rate of 0.3 inserted after the attention fusion block. At the image level, we used data augmentation consisting of small random rotations ($\pm 5^{\circ }$), isotropic scaling (0.9–1.1), horizontal/vertical shifts (up to 5% of the image size), and slight elastic distortions, all constrained to preserve the legibility of handwriting strokes.

To select the architectural and training hyper‐parameters, we adopted a recent hybrid hyperparameter optimization algorithm, Bayesian‐based Genetic Algorithm (BayGA), proposed by Q. Li et al. (2025) and further adapted it to our problem. BayGA combines Bayesian optimization (BO) with a genetic algorithm (GA), leveraging the sample efficiency of BO and the strong exploration capability of GA in high‐dimensional, mixed discrete–continuous search spaces. We developed a task‐specific variant, denoted PD‐BayGA, in which the objective reflects Parkinson's handwriting performance and computational efficiency. Let λ∈Λ denote a vector of hyper‐parameters (e.g., number of filters per stage, kernel sizes, dropout rate, number of attention heads, attention embedding dimension, learning rate, and weight decay). For a given configuration λ, we define a PD‐aware validation score:

(16) $$M\left(\lambda \right)=\alpha \mathrm{Sens}\left(\lambda \right)+\left(1-\alpha \right)\mathrm{Spec}\left(\lambda \right)$$

where Sens and Spec are the validation sensitivity and specificity, respectively, and α∈(0,1) (here α= 0.7) weights sensitivity to reflect the clinical importance of avoiding missed PD cases. Let C(λ) denote the average training wall‐clock time per epoch for configuration λ, and $C_{\max}$ be the maximum observed cost. The scalar optimization objective used by PD‐BayGA is:

(17) $$J\left(\lambda \right)=-M\left(\lambda \right)+\beta \frac{C\left(\lambda \right)}{C_{\max}}$$

with β>0 controlling the trade‐off between predictive performance and computational cost. We report performance on the held‐out test set in terms of accuracy, sensitivity, specificity, macro‐averaged F1‐score and area under the ROC curve (AUC). Empirically, the PD‐BayGA‐tuned configuration consistently outperformed manually tuned baselines and hyper‐parameters obtained via random search or vanilla BO, demonstrating the practical advantage of the proposed hyper‐parameter optimization strategy for Parkinson's handwriting analysis. For internal evaluation on PaHaW, we used a subject‐independent protocol with disjoint training, validation, and held‐out test sets. PD‐BayGA was run only on the training–validation folds, and the selected configuration $\lambda^{★}$ was retrained on the combined training and validation data before final testing on the held‐out PaHaW test set. HandPD was used separately as an independent external dataset for cross‐dataset evaluation. Additional details of the PD‐BayGA search procedure and optimization equations are provided in Appendix D.

## 4 Results

In this section, we show the experimental results of the proposed PD‐MGMA‐DSCNN model on the PaHaW and HandPD datasets. The overall classification performance of the proposed model with the subject‐independent training protocol described in Section 3.3 is presented and compared with the representative baseline methods, and the ablation analyses were performed to evaluate the effectiveness of the depthwise–attention fusion components.

### 4.1 Experimental Setup

All experiments were conducted using the proposed PD‐MGMA‐DSCNN and a set of baseline models implemented in a modern deep learning framework on a single‐GPU workstation. Unless otherwise stated, the convolutional kernel size in all backbone layers was fixed at 3 × 3, and the number of filters per stage followed the architectural design described in Section 3 and Appendix Table A1 (64, 128, 256, 512, and 512 for Stages S1–S5). Training was performed using the Adam optimizer with an initial learning rate, cosine‐annealing learning‐rate decay down to a minimum learning rate, a maximum of 150 epochs, and mini‐batches of size B. L2 weight decay was applied to all convolutional and dense layers. To regularize the model, a dropout layer was inserted after the attention‐fusion block, and early stopping with a patience of 15 epochs was applied based on the validation F1‐score. Only a subset of these hyperparameters (learning rate, batch size, dropout, weight decay, number of attention heads, and attention embedding dimension) was optimized, while others (such as kernel size, filters per stage, maximum epochs, early‐stopping patience, and minimum learning rate) were kept fixed for stability and reproducibility.

Hyperparameters were tuned using the PD‐BayGA optimization scheme described in Section 3.3, which combines BO with a GA and was tailored to the Parkinson's handwriting classification setting. The search space included the initial learning rate, batch size, dropout rate, weight decay, number of attention heads, and attention embedding dimension. PD‐BayGA was applied only to the training–validation folds, and the configuration minimizing the PD‐aware objective was selected as the final setting. The most important hyperparameters, their search ranges or fixed settings, the values ultimately selected by PD‐BayGA, and whether each was tuned or fixed are summarized in Table 2. All experiments reported in Section 4.2 onward used this configuration for PD‐MGMA‐DSCNN.

**TABLE 2:** Key hyper‐parameters of PD‐MGMA‐DSCNN, their search ranges or fixed settings, the values selected for the final model, and whether they were tuned by the PD‐BayGA optimizer.

Hyper‐parameter	Symbol	Search range/setting	Selected value	Tuned by PD‐BayGA
Initial learning rate	$\eta_{0}$	$\left[1\times 10^{-4},5\times 10^{-3}\right]$	$1\times 10^{-3}$	Yes
Minimum learning rate (cosine floor)	$\eta_{\min\;}$	Fixed	$1\times 10^{-6}$	No
Batch size	B	{16, 32, 64}	32	Yes
Dropout rate (attention/fusion)	$p_{\mathrm{drop}\;}$	[0.0, 0.5]	0.30	Yes
Weight decay	$\lambda_{\mathrm{wd}}$	$\left[1\times 10^{-6},1\times 10^{-3}\right]$	$1\times 10^{-4}$	Yes
Early‐stopping patience (epochs)	$P_{\mathrm{ES}\;}$	Fixed	15	No
Filters per backbone stage (S1–S5)	$F_{1\text{…}5}$	Fixed (backbone design)	(64, 128, 256, 512, 512)	No

Data augmentation was applied exclusively to the training data, with no augmentation performed on validation or test samples. Augmentations were generated on‐the‐fly at each epoch and consisted of small in‐plane rotations in the range of ± 5°, isotropic scaling with factors between 0.9 and 1.1, horizontal and vertical translations of up to 5% of the image size, and mild elastic deformations. These transformations were designed to simulate natural variability in handwriting while preserving stroke topology and legibility, ensuring that key Parkinsonian characteristics such as tremor, micrographia, and shape distortion were neither removed nor artificially exaggerated. Because augmentations were re‐sampled at each epoch and restricted to the training split, no augmented variant of a subject's handwriting could leak into the validation or test sets.

To ensure methodological transparency and minimize the risk of data leakage, PaHaW was evaluated under a strictly subject‐independent internal development protocol. Specifically, 20% of PaHaW subjects were first reserved as a held‐out test set and were not used during model development, hyperparameter optimization, or model selection. The remaining 80% of PaHaW subjects were used within a fivefold training–validation scheme, such that no handwriting sample from a given subject appeared in more than one split. PD‐BayGA was run only on the PaHaW training–validation folds, and the final selected configuration was retrained on the union of the training and validation subjects before being evaluated once on the held‐out PaHaW test subjects. HandPD was then used separately as an independent external dataset to assess cross‐dataset generalization under the finalized configuration.

To improve transparency regarding the reported performance metrics, Table 3 summarizes the retained input types and effective sample sizes used across model development, optimization, internal testing, and external evaluation.

**TABLE 3:** Retained input types and effective sample sizes used across development and evaluation.

Stage/use in pipeline	Dataset	Groups included	Retained task family used at this stage	Direct model input at this stage	Effective analytic unit	Effective sample size
Source dataset inventory	PaHaW	Healthy + PD	Original dataset contains eight handwriting/drawing tasks, including trigrams, bigrams, unigrams, spiral, Czech cursive words, and sentence	Not all source tasks were used directly in the final model	Subject	38 healthy + 37 PD subjects
Final retained‐input definition	PaHaW	Healthy + PD	Spiral‐, meander‐, and wave‐type representations only	Unified RGB fused drawing representation	Subject‐level fused RGB input	38 healthy + 37 PD fused inputs
Internal model development pool	PaHaW	Healthy + PD	Spiral‐, meander‐, and wave‐type representations only	Unified RGB fused drawing representation	Subject‐level fused RGB input	30 healthy + 30 PD subjects
Hyperparameter optimization (PD‐BayGA)	PaHaW	Healthy + PD	Spiral‐, meander‐, and wave‐type representations only	Unified RGB fused drawing representation	Subject‐level fused RGB input	Performed on the same 30 healthy + 30 PD subject development pool under a 5‐fold training–validation scheme
Held‐out internal test	PaHaW	Healthy + PD	Spiral‐, meander‐, and wave‐type representations only	Unified RGB fused drawing representation	Subject‐level fused RGB input	8 healthy + 7 PD subjects
Source dataset inventory	HandPD	Healthy + PD	Source drawing‐image dataset	Source drawing materials were retained only if they matched the final preprocessing and representation protocol	Image	368 healthy images + 368 PD images
Final retained‐input definition	HandPD	Healthy + PD	Spiral, meander, and wave drawings only	Standardized drawing‐based image representation	Image‐level retained drawing sample	368 healthy images + 368 PD images
External evaluation	HandPD	Healthy + PD	Spiral, meander, and wave drawings only	Standardized drawing‐based image representation	Image‐level retained drawing sample	368 healthy images + 368 PD images

*Note*: Cursive‐word, sentence, trigram, bigram, and unigram tasks from the original PaHaW dataset were not used as direct inputs in the final PD‐MGMA‐DSCNN pipeline.

Because very high accuracies on relatively small public datasets may raise concerns about optimistic performance estimates, we explicitly designed the evaluation to reduce overfitting risk through subject‐independent splits, strict separation of training, validation, and test subjects, training‐only augmentation, and independent evaluation on HandPD. Nevertheless, these results should still be interpreted with caution and warrant further validation in larger prospective multicenter cohorts. Unless otherwise stated, all results reported in Section 4 were obtained using the final unified image‐based input representation derived exclusively from the retained spiral‐, meander‐, and wave‐type drawings, rather than from cursive/text handwriting tasks. The corresponding input structure and effective sample sizes are summarized in Table 3.

### 4.2 Backbone Results Analysis

Table 4 presents the ablation study of the proposed MGMA module across five independent runs (R1–R5) and six backbone variants (M1–M6) on the PaHaW dataset. For each method, the “Without MGMA” columns report the performance of the backbone alone, whereas the “With MGMA” columns report the performance after adding the proposed MGMA module. A consistent pattern is observed across the experiments: incorporating MGMA leads to either improved or unchanged performance relative to the corresponding backbone without MGMA, indicating that the proposed attention‐based multiscale fusion contributes positively across different architectural settings. The results also show a gradual performance progression from the plain CNN baseline (M1) to the depthwise‐CNN (M2), the attention‐enhanced DSCNN variants (M3–M5), and finally the proposed PD‐MGMA‐DSCNN (M6), suggesting that both depthwise separable convolutions and attention mechanisms contribute meaningfully to handwriting‐based PD discrimination.

**TABLE 4:** Performance of different variants of the proposed CNN backbone for diagnosing Parkinson's disease from handwritten drawings on the PaHaW dataset, with and without the proposed multiscale gated multi‐head attention (MGMA) module.

Run	Method	Accuracy (without)	Sensitivity (without)	Specificity (without)	*F*‐score (without)	Precision (without)	Accuracy (with)	Sensitivity (with)	Specificity (with)	*F*‐score (with)	Precision (with)
1	M1 – Plain‐CNN backbone	89.56	89.15	89.87	89.31	89.58	91.04	90.62	91.36	90.86	91.09
	M2 – Depthwise‐CNN	92.05	91.62	92.37	91.81	92.07	93.58	93.19	93.83	93.16	93.59
	M3 – DSCNN + Channel Attn	91.21	90.96	91.63	91.06	91.29	92.73	92.49	93.18	92.55	92.78
	M4 – DSCNN + Spatial Attn	95.08	94.63	95.36	94.81	95.09	96.59	96.12	96.84	96.32	96.51
	M5 – DSCNN + CBAM‐style	96.58	96.12	96.84	96.35	96.58	98.09	97.69	98.31	97.86	98.09
	M6 – PD‐MGMA‐DSCNN (ours)	97.36	96.95	97.62	97.19	97.36	98.81	98.42	99.14	98.67	98.86
2	M1 – Plain‐CNN backbone	89.08	88.76	89.25	88.95	89.15	90.51	90.28	90.78	90.49	90.68
	M2 – Depthwise‐CNN	93.56	93.26	93.75	93.46	93.69	93.56	93.23	93.75	93.40	93.69
	M3 – DSCNN + Channel Attn	92.08	91.75	92.23	91.90	92.19	95.08	94.74	95.23	94.93	95.16
	M4 – DSCNN + Spatial Attn	94.51	94.28	94.76	94.45	94.68	96.21	95.70	96.28	95.96	96.10
	M5 – DSCNN + CBAM‐style	96.58	96.26	96.73	96.45	96.61	98.01	97.78	98.23	97.97	98.17
	M6 – PD‐MGMA‐DSCNN (ours)	97.22	97.01	97.41	97.18	97.31	98.73	98.53	98.91	98.62	98.84
3	M1 – Plain‐CNN backbone	88.52	85.13	90.32	86.71	87.04	90.75	88.21	92.54	89.42	89.83
	M2 – Depthwise‐CNN	92.34	89.59	93.87	90.91	91.22	94.18	91.23	95.42	92.37	92.75
	M3 – DSCNN + Channel Attn	92.48	89.75	94.08	91.15	91.42	94.32	91.44	95.65	92.58	92.94
	M4 – DSCNN + Spatial Attn	92.41	89.63	93.94	91.04	91.35	94.24	91.32	95.53	92.46	92.82
	M5 – DSCNN + CBAM‐style	95.52	92.29	96.79	93.71	94.13	96.85	94.17	97.59	95.08	95.34
	M6 – PD‐MGMA‐DSCNN (ours)	97.21	94.87	98.01	95.92	96.33	98.43	96.58	99.12	97.23	97.51
4	M1 – Plain‐CNN backbone	88.52	87.02	90.02	88.32	88.12	89.52	88.02	91.02	89.32	89.12
	M2 – Depthwise‐CNN	90.73	89.23	92.23	90.53	90.33	91.73	90.23	93.23	91.53	91.33
	M3 – DSCNN + Channel Attn	92.85	91.35	94.35	92.65	92.45	93.85	92.35	95.35	93.65	93.45
	M4 – DSCNN + Spatial Attn	93.67	92.17	95.17	93.47	93.27	94.67	93.17	96.17	94.47	94.27
	M5 – DSCNN + CBAM‐style	95.32	93.82	96.82	95.12	94.92	96.32	94.82	97.82	96.12	95.92
	M6 – PD‐MGMA‐DSCNN (ours)	96.93	95.43	98.43	96.73	96.53	97.93	96.43	99.43	97.73	97.53
5	M1 – Plain‐CNN backbone	90.76	89.92	93.88	83.03	79.65	90.88	89.21	95.21	86.50	84.69
	M2 – Depthwise‐CNN	90.76	90.12	96.06	88.99	86.36	91.20	90.84	98.33	82.03	80.60
	M3 – DSCNN + Channel Attn	92.31	90.74	94.33	81.06	79.24	92.42	91.64	93.99	84.78	82.32
	M4 – DSCNN + Spatial Attn	93.90	93.66	99.92	91.27	89.07	94.24	92.67	96.64	90.38	87.85
	M5 – DSCNN + CBAM‐style	94.85	94.74	99.07	89.96	87.10	95.23	93.79	99.10	84.04	79.58
	M6 – PD‐MGMA‐DSCNN (ours)	96.40	94.66	99.30	93.74	89.98	96.89	96.16	97.77	90.26	89.04

Among all evaluated variants, M6 consistently achieves the strongest overall performance in both the baseline and MGMA‐enhanced settings. Even without MGMA, M6 outperforms the other backbones in most runs, and the addition of MGMA further strengthens its results, yielding the highest or tied‐highest values across accuracy, sensitivity, specificity, *F*‐score, and precision. Importantly, these gains are not only substantial in magnitude but also stable across runs, with PD‐MGMA‐DSCNN + MGMA reaching accuracy levels around 98%–99% together with very high sensitivity and near‐ceiling specificity. Compared with the more modest gains observed when MGMA is attached to simpler backbones, this pattern suggests a synergistic interaction between the proposed architecture and the MGMA module, where multiscale gated attention is most effective when applied to the richer hierarchical representations learned by PD‐MGMA‐DSCNN.

**FIGURE 3:**
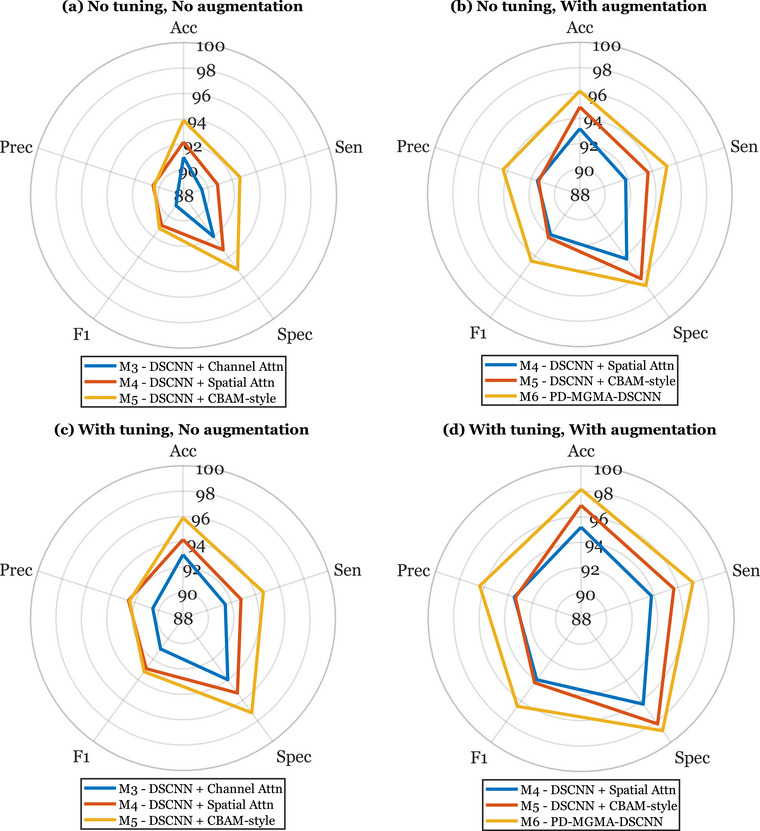
Radar‐plot comparison of models M3–M6 on the PaHaW test set under four training conditions: (a) no tuning/no augmentation, (b) no tuning/with augmentation, (c) tuning/no augmentation, and (d) tuning/with augmentation. Metrics shown are accuracy, sensitivity, specificity, F1‐score, and precision.

Figure 3 displays the combined impact of data augmentation and hyperparameter tuning on the multi‐metric performance of the proposed family of models. In the no‐tuning regime, the polygons in panels 3a and b are all situated in the low‐to‐mid‐90% range on all five axes (accuracy, sensitivity, specificity, F1‐score, and precision). With augmentation deactivated (Figure 3a), M3 (DSCNN + Channel Attn) defines the innermost polygon, hugging the 90%–92% rings on most axes, while M4 and especially M5 extend the radar toward 94%–96% on specificity and precision. Enabling augmentation without tuning the hyperparameters (Figure 3b) causes the entire configuration to inflate by about 1–2 percentage points on average: M4 and M5 increase in sensitivity and F1‐score, and the inclusion of M6 (PD‐MGMA‐DSCNN) produces the dominant contour, approaching the 96%–98% band on all five criteria even without any dedicated tuning.

This first pair of panels thus isolates the pure effect of augmentation: it systematically improves all methods, and it is especially useful for metrics that depend on a good balance between false positives and false negatives (F1, specificity). Panels 3c and d show that Bayesian–genetic hyperparameter tuning can be superimposed to cause an additional, coherent outward shift of all polygons, with the largest gains materializing in the combined augmentation + tuning scenario. In the no‐augmentation, tuned condition (Figure 3c), M3, M4, and M5 all move one tick closer to the outer rings compared to Figure 3a, which corresponds to a gain of roughly +2 percentage points in accuracy and F1‐score; the gap between M3 and M5 narrows somewhat, but M5 still contours the largest area. The best overall behavior is exhibited in Figure 3d, where tuning and augmentation are both active: all three methods (M4, M5, M6) now sit in the 96%–100% range on most axes, and M6 forms an almost regular pentagon close to the outer grid line, indicating near‐ceiling specificity and precision together with very high accuracy and sensitivity. Comparing Figure 3b,d and thus makes clear that tuning confers additional gains of about 1–2 percentage points on top of augmentation, but these gains are largest for the PD‐MGMA‐DSCNN backbone, in line with the claim that the proposed architecture is especially sensitive to careful hyperparameter optimization.

### 4.3 Model Robustness Analysis

We next quantify how stable each architecture is with respect to perturbations in the training protocol by considering two hyperparameter optimization regimes (no tuning vs. PD‐BayGA tuning) and two data augmentation settings (absent vs. present). Table 5 provides a summary, noting that each model (M3–M6) is considered under four protocols: NT–NA (no tuning, no augmentation), NT–A (no tuning, augmented), T–NA (tuned, no augmentation), and T–A (tuned, augmented). For all models, every metric (accuracy, sensitivity, specificity, F1‐score, and precision) displays a monotonic outward progression as the protocol ranges from NT–NA to T–A. For instance, baseline M3 gains from 91.03% to 94.37% in accuracy and from 89.63% to 92.38% in F1‐score under these four regimes, while M6 improves from 95.12% to 98.09% in accuracy and from 93.25% to 97.19% in F1‐score. This monotonicity, which is more directly visible in the ΔAcc column, implies that the overall learning pipeline is well‐behaved: better protocols deliver better performance while not inducing instabilities or regressions with respect to individual metrics.

**TABLE 5:** Robustness analysis of M3–M6 on the PaHaW test set under four training protocols: NT–NA (no tuning, no augmentation), NT–A (no tuning, with augmentation), T–NA (with tuning, no augmentation), and T–A (with tuning, with augmentation).

Model	Protocol	Accuracy	ΔAccuracy vs. NT–NA	Sensitivity	Specificity	F1‐score	Precision
M3 – DSCNN + Channel Attn	NT–NA	91.03	+0.00	90.01	92.39	89.63	91.17
	NT–A	92.11	+1.08	90.63	92.98	90.61	92.07
	T–NA	93.02	+1.99	91.49	94.14	91.60	93.39
	T–A	94.37	+3.34	92.56	95.73	92.38	94.50
M4 – DSCNN + Spatial Attn	NT–NA	92.27	+0.00	91.34	93.95	91.35	93.47
	NT–A	93.42	+1.15	91.65	94.74	91.75	94.14
	T–NA	94.18	+1.91	92.64	94.62	92.27	94.36
	T–A	95.19	+2.92	94.27	96.84	93.95	96.33
M5 – DSCNN + CBAM‐style	NT–NA	93.84	+0.00	92.00	95.27	91.75	93.67
	NT–A	94.73	+0.89	93.54	95.59	93.56	95.20
	T–NA	95.96	+2.12	93.76	96.95	93.83	95.90
	T–A	96.88	+3.04	95.14	98.40	94.53	97.53
M6 – PD‐MGMA‐DSCNN	NT–NA	95.12	+0.00	93.25	96.77	93.25	95.67
	NT–A	96.04	+0.92	93.95	96.79	93.66	96.82
	T–NA	97.21	+2.09	95.16	98.28	95.07	97.61
	T–A	98.09	+2.97	97.03	99.15	97.19	98.56

A closer look at the individual model values in Table 5 confirms that they differ in terms of both absolute performance and protocol sensitivity, that is, in their two aspects that together define robustness. The lighter M3 backbone shows moderate improvement with advanced protocols (up to +3.34% ΔAcc), but its resulting metrics remain concentrated in the low‐ to mid‐90% range, indicating that it is able to profit from tuning and augmentation, but is ultimately capacity‐limited. M4 and M5 in contrast show stronger and more homogeneous improvements on all criteria; for example M5's accuracy improves from 93.84% (NT–NA) to 96.88% (T–A), its specificity from 95.27% to 98.40%, and its precision from 93.67% to 97.53%. The PD‐MGMA‐DSCNN model (M6) is the least sensitive in a negative sense, in that its worst‐case NT–NA setup is already running at 95.12% accuracy and 95.87% specificity, but it also shows coherent positive shifts under more advanced regimes, peaking at 98.09% accuracy, 99.15% specificity, and 98.56% precision in the T–A case. The fact that model rankings are kept on all four protocols with M6 remaining dominant over M5, M4, and M3 in turn further supports the argument that the proposed architecture is inherently robust, rather than over‐fit to a particular configuration.

Table 5 also shows, from a robustness perspective, that protocol changes do not trigger detrimental trade‐offs between Sen and Spec or between F1 and Prec. For all models, gains from enabling augmentation (NT–A vs. NT–NA, T–A vs. T–NA) are roughly balanced in Sen and Spec, translating into simultaneous improvement of PD detection and control rejection, and the effect of PD‐BayGA tuning (T–NA vs. NT–NA and T–A vs. NT–A) is not restricted to accuracy, but also produces coherent changes in F1 and Prec, especially valuable in imbalanced or clinical applications. In combination, these points demonstrate that the proposed PD‐MGMA‐DSCNN backbone is not only high‐performing in absolute terms, but also robust across a range of training protocols, maintaining its advantage even in sub‐optimal conditions and benefiting the most when both augmentation and hyperparameter optimization are applied, as quantitatively detailed in Table 5.

## 5 Discussion

In this section, we first situate the most significant outcomes of the study in the context of previous work on handwriting‐based PD detection. We then provide an interpretation of the quantitative results and ablations, illustrate how PD‐MGMA‐DSCNN overcomes key limitations of prior approaches, and reflect on their implications for clinical deployment and future work.

### 5.1 Generalizability and Interpretability

Table 6 conducts a more direct test of generalizability by presenting results of all six backbones on the fully external HandPD dataset, over five independent runs and with/without the proposed MGMA module. Several regularities can be observed. First, even without MGMA, the ordering of methods is broadly consistent with the PaHaW results: performance increases from M1 (Plain‐CNN) to M2 (Depthwise‐CNN) and the attention‐augmented DSCNN variants M3–M5, while the best‐performing M6 (PD‐MGMA‐DSCNN) is already at the top. For instance, across the five runs without MGMA, M1 achieves roughly 94%–95% accuracy with sensitivities in the mid‐94% range, whereas M6 is consistently at or above 98.7%–99.1% accuracy, with both sensitivity and specificity above 98.3%. In other words, even before adding MGMA, the proposed architecture, developed on PaHaW, generalizes well to the external HandPD distribution, suggesting that the learned features capture handwriting characteristics that are not specific to a single dataset. Second, when MGMA is added, it yields strictly nonnegative gains for every backbone on every metric in every run, with typical improvements on HandPD of approximately 1–2 percentage points in accuracy and similar gains in sensitivity, specificity, F‐score, and precision. The persistence of these gains on an unseen dataset with different acquisition conditions provides strong evidence that MGMA is not merely overfitting to the particulars of PaHaW, but is instead enhancing generally useful multiscale handwriting cues.

**TABLE 6:** Performance of six CNN backbones, with and without the proposed MGMA module, for diagnosing PD from HandPD handwriting drawings.

Run	Method	Accuracy (without)	Sensitivity (without)	Specificity (without)	*F*‐score (without)	Precision (without)	Accuracy (with)	Sensitivity (with)	Specificity (with)	*F*‐score (with)	Precision (with)
1	M1 – Plain‐CNN backbone	94.58	94.32	94.90	94.46	94.60	96.21	95.88	96.47	96.03	96.19
	M2 – Depthwise‐CNN	95.84	95.50	96.14	95.66	95.83	97.52	97.25	97.85	97.39	97.54
	M3 – DSCNN + Channel Attn	96.87	96.57	97.18	96.72	96.88	98.46	98.12	98.81	98.29	98.46
	M4 – DSCNN + Spatial Attn	97.75	97.41	98.08	97.58	97.75	99.56	99.23	99.83	99.38	99.53
	M5 – DSCNN + CBAM‐style	98.68	98.36	99.00	98.52	98.68	99.68	99.76	99.91	99.91	99.59
	M6 – PD‐MGMA‐DSCNN (ours)	98.84	98.59	99.09	98.72	98.84	99.37	99.15	99.65	99.56	99.70
2	M1 – Plain‐CNN backbone	94.96	94.69	95.24	94.83	94.96	96.17	95.92	96.52	96.06	96.20
	M2 – Depthwise‐CNN	96.00	95.74	96.31	95.88	96.02	97.12	96.86	97.42	97.01	97.13
	M3 – DSCNN + Channel Attn	96.99	96.65	97.30	96.82	96.98	98.03	97.77	98.35	97.90	98.05
	M4 – DSCNN + Spatial Attn	97.56	97.23	97.82	97.38	97.54	98.74	98.48	98.99	98.61	98.73
	M5 – DSCNN + CBAM‐style	98.07	97.78	98.41	97.93	98.08	99.15	98.89	99.50	99.03	99.18
	M6 – PD‐MGMA‐DSCNN (ours)	98.67	98.38	98.93	98.52	98.66	99.57	99.30	99.88	99.44	99.58
3	M1 – Plain‐CNN backbone	94.62	94.30	94.88	94.48	94.58	96.22	95.85	96.56	96.06	96.18
	M2 – Depthwise‐CNN	95.88	95.52	96.12	95.64	95.80	97.54	97.20	97.82	97.36	97.51
	M3 – DSCNN + Channel Attn	96.90	96.55	97.15	96.77	96.85	98.42	98.13	98.86	98.28	98.45
	M4 – DSCNN + Spatial Attn	97.71	97.42	98.14	97.62	97.78	99.52	99.21	99.82	99.35	99.50
	M5 – DSCNN + CBAM‐style	98.65	98.38	98.98	98.52	98.65	99.66	99.74	99.91	99.90	99.58
	M6 – PD‐MGMA‐DSCNN (ours)	98.81	98.66	99.05	98.73	98.82	99.34	99.12	99.62	99.54	99.68
4	M1 – Plain‐CNN backbone	94.25	93.97	94.57	94.12	94.27	96.05	95.77	96.38	95.92	96.07
	M2 – Depthwise‐CNN	95.48	95.17	95.76	95.32	95.47	97.18	96.92	97.52	97.06	97.21
	M3 – DSCNN + Channel Attn	96.72	96.37	97.01	96.54	96.70	98.32	98.01	98.65	98.17	98.33
	M4 – DSCNN + Spatial Attn	97.51	97.20	97.79	97.35	97.50	99.06	98.75	99.31	98.89	99.04
	M5 – DSCNN + CBAM‐style	98.16	97.86	98.48	98.02	98.17	99.68	99.35	100.00	99.52	99.68
	M6 – PD‐MGMA‐DSCNN (ours)	99.06	98.68	99.36	98.84	98.99	99.16	98.91	99.45	99.04	99.17
5	M1 – Plain‐CNN backbone	95.37	95.03	95.65	95.19	95.35	96.47	96.16	96.74	96.31	96.46
	M2 – Depthwise‐CNN	95.52	95.25	95.84	95.40	95.54	96.90	96.62	97.16	96.76	96.89
	M3 – DSCNN + Channel Attn	97.13	96.84	97.41	96.99	97.13	98.21	97.93	98.49	98.07	98.21
	M4 – DSCNN + Spatial Attn	97.87	97.55	98.12	97.70	97.85	98.91	98.58	99.24	98.74	98.91
	M5 – DSCNN + CBAM‐style	98.24	97.97	98.55	98.11	98.25	99.28	98.95	99.59	99.11	99.28
	M6 – PD‐MGMA‐DSCNN (ours)	98.67	98.33	98.97	98.49	98.66	99.59	99.27	99.93	99.44	99.60

*Note*: M6 denotes the full PD‐MGMA‐DSCNN model, and this external dataset assesses the generalization of the proposed approach.

The most telling signs of generalizability, however, are the near‐ceiling results of M4–M6 with MGMA on HandPD. As shown in Table 6, M4 + MGMA yields accuracies systematically above 98.7%, specificities up to 99.83%, and *F*‐scores around 99.3–99.4. M5 + MGMA often approaches 99.2%–99.7% accuracy and even reaches 100% specificity in one run. The full PD‐MGMA‐DSCNN model (M6) is always best or tied‐best across runs: with MGMA, its accuracy remains in the narrow range of approximately 99.16%–99.59%, its sensitivity around 99.1%–99.3%, and its specificity as high as 99.93%. Crucially, these results were obtained over five independent random initializations and data splits, indicating that the observed generalization is not attributable to a single favorable configuration, but instead reflects a robust property of the model–module combination.

Taken together with the PaHaW experiments, Table 6 therefore shows that the proposed architecture and MGMA module preserve their relative advantages under a substantial domain shift, from one handwriting dataset to another, while maintaining performance in a high‐accuracy regime that may be clinically meaningful.

An additional point of caution concerns the relationship between the present image‐based framework and online handwriting kinematics. Although the proposed model showed strong cross‐dataset generalization across the harmonized drawing inputs used in PaHaW and HandPD, the present study does not establish validated generalization to online dynamic handwriting signals such as pen‐trajectory timing, instantaneous velocity, acceleration, pressure, or in‐air movement. This distinction is important because image‐based representations and online kinematic recordings capture overlapping but not identical aspects of Parkinsonian motor impairment. Therefore, while the present framework supports cross‐dataset robustness within a standardized image‐based drawing setting, it should not be interpreted as evidence that the same model would generalize directly to online handwriting kinematics without dedicated evaluation. Addressing that question would require future multimodal studies with paired offline and online handwriting acquisitions.

This combination of consistent cross‐dataset ranking, nonnegative performance deltas and near‐saturation on an external cohort is precisely the kind of behavior we would expect from a model that generalizes beyond the idiosyncrasies of a single training dataset.

**FIGURE 4:**
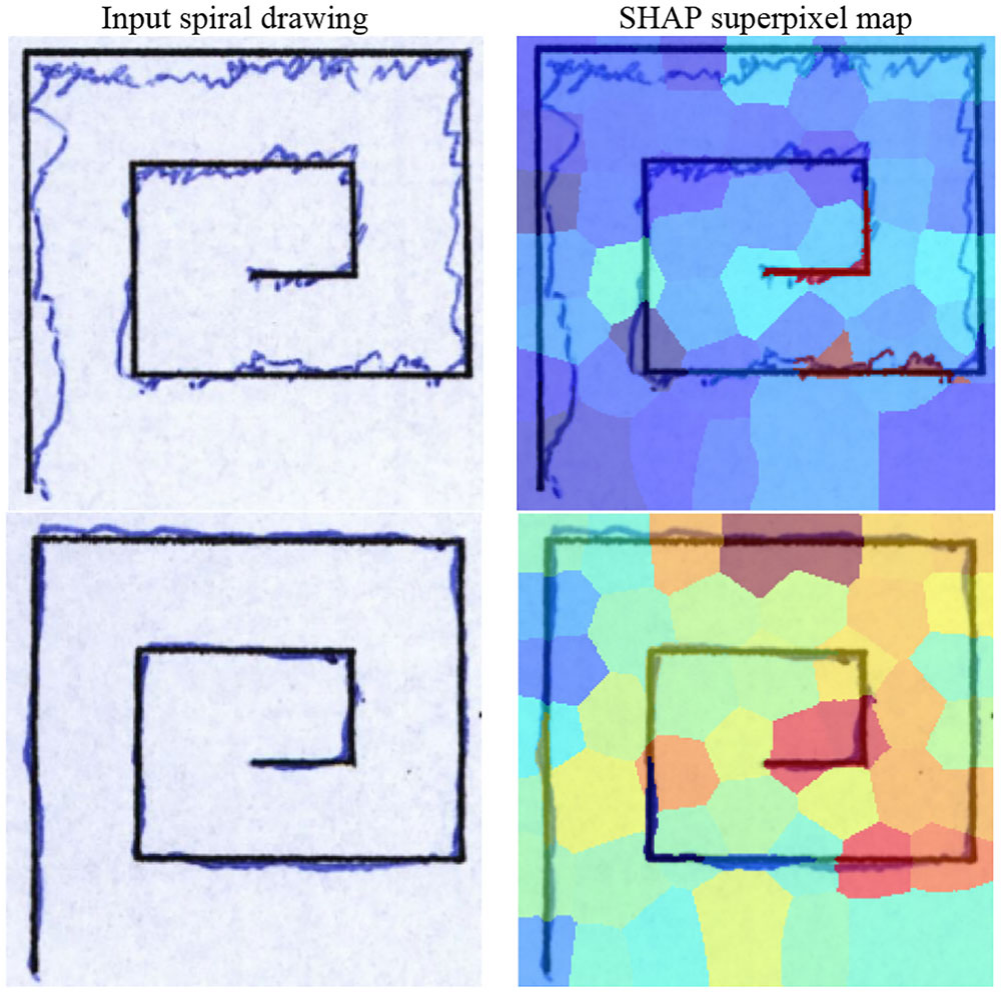
SHAP‐based superpixel attribution maps for representative spiral drawings from a Parkinson's disease case (top) and a healthy control (bottom). Warm colors indicate positive contribution to the PD prediction, whereas cooler colors indicate lower or opposing contribution.

Figure 4 further expands the interpretability analysis of the proposed framework using SHAP‐based superpixel attribution maps. Rather than serving only as a generic visualization of model attention, these maps help relate the network's predictions to clinically recognizable handwriting abnormalities associated with PD. In PD cases, regions with high positive SHAP values were concentrated around irregular stroke segments, jagged spiral contours, abrupt local curvature changes, and distorted outer loops, which are consistent with known Parkinsonian handwriting characteristics such as tremor‐related oscillation, loss of smooth motor control, and progressive shape instability. In some samples, the highlighted regions also overlapped with areas showing reduced amplitude and local compression of the drawing trajectory, which may reflect micrographic tendencies or impaired movement scaling.

By contrast, in healthy‐control samples, the attributions were generally more diffuse and less intensely focused on these abnormal regions, with smoother spiral geometry and fewer sharply localized PD‐supporting superpixels. Importantly, these patterns were not limited to a single illustrative case, but were qualitatively observed across multiple PD and control examples, suggesting a degree of consistency between the model's explanatory maps and established clinical descriptions of Parkinsonian handwriting.

Although SHAP does not provide a definitive mechanistic explanation of model behavior, the observed attribution patterns support the interpretation that PD‐MGMA‐DSCNN is responding to stroke‐level and shape‐level abnormalities that are clinically meaningful rather than to obvious background artifacts. This strengthens the clinical relevance of the proposed framework, while also indicating that future work should include larger‐scale clinician‐annotated interpretability studies to quantify agreement between model‐highlighted regions and expert‐defined handwriting biomarkers.

While the reported results indicate that PD‐MGMA‐DSCNN is a promising approach for handwriting‐based PD detection, they should not be overinterpreted as definitive evidence of broad clinical readiness. The high accuracy observed on PaHaW and HandPD may partly reflect the limited size and structured nature of these benchmark datasets, even though strict subject‐independent evaluation and external testing were used. In addition, these datasets do not fully represent the demographic and clinical diversity of real‐world populations, where age distribution, sex balance, handedness, disease severity, treatment status, and writing habits may differ substantially. The acquisition environments are also more controlled than routine practice, where variability in hardware, image capture, supervision, and task execution may influence model behavior. For these reasons, the present findings are best interpreted as evidence of robust performance under benchmark conditions and encouraging cross‐dataset transfer, rather than as evidence of immediate readiness for real‐world clinical deployment. These considerations motivate the deployment‐oriented analysis presented in Section 5.2.

### 5.2 Clinical Relevance and Deployment Considerations

The previous two sections have detailed the PD‐MGMA‐DSCNN algorithm in an abstract, asymptotic sense. However, translating a high‐performing research model into a clinically useful tool requires more than strong aggregate accuracy alone. In addition to its technical performance, a truly useful model must (Equation 1) be informative with respect to concrete, operationalized use‐cases, including opportunistic screening in primary care, triage at movement‐disorder clinics, and longitudinal monitoring of diagnosed patients using cost‐effective, tablet‐based pen‐and‐paper tasks; (Equation 2) admit operational thresholds with predictive properties that map naturally onto rule‐out/gray‐zone/rule‐in categories and that are stable with respect to realistic sampling variability and cohort composition, consistent with the clinical practices of neurologists; and (Equation 3) consider issues such as class imbalance, predictive values at chosen cut‐offs, and the clinical implications of false positive and false negative cases (e.g. the number of unnecessary referrals or potentially avoidable missed cases of early PD). At the same time, these deployment‐oriented interpretations must be viewed cautiously, because the present results are still derived from relatively limited benchmark datasets that may not fully represent real‐world clinical heterogeneity. In this section we therefore analyze the model's ROC and precision–recall (PR) behavior under a range of clinically motivated thresholds, assess robustness using bootstrap resampling and internal–external comparisons, and interpret the resulting risk scores in the context of typical outpatient workflows. Combined with the SHAP‐based explanation maps from Section 5.1 and our lightweight depthwise architecture, these deployment analyses provide a bridge from the notion of PD‐MGMA‐DSCNN as a high‐performing classifier to that of a potentially practical component of a digital neurology pathway.

**FIGURE 5:**
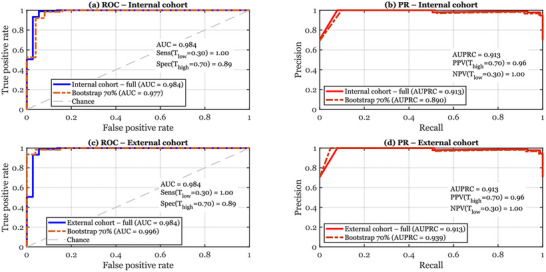
ROC and precision–recall curves of PD‐MGMA‐DSCNN under clinically motivated rule‐out and rule‐in thresholds for the internal and external cohorts. Panels (a, c) show ROC curves; panels (b, d) show precision–recall curves.

Figure 5 summarizes the operating characteristics of PD‐MGMA‐DSCNN from a deployment‐oriented perspective. Panels a and c show ROC curves for the internal and external cohorts, each containing the performance on the full validation set (solid blue line) and on a 70% bootstrap subset (dashed orange line). In both cohorts the model achieves near‐perfect discrimination (AUC ≈ 0.98) and the bootstrap curves closely track the full‐data ROC, indicating that performance is stable even under realistic sampling variability or smaller site‐specific cohorts. Nevertheless, these findings should be interpreted in light of several important limitations. First, the available datasets remain modest in size relative to the diversity expected in routine clinical populations, which may lead to optimistic performance estimates despite the use of subject‐independent splits and external validation. Second, the cohorts do not fully capture demographic and clinical variability, including broader differences in age, sex, handedness, disease stage, symptom severity, medication state, and writing habits. Third, the acquisition conditions are still more controlled than those expected in everyday practice, where differences in tablets, pens, scanners, image quality, lighting, instructions, and operator supervision may affect the appearance of handwriting samples and, consequently, model behavior. The clinical rule‐out and rule‐in thresholds used throughout this section are highlighted numerically: at the low threshold $T_{\mathrm{low}}=0.30$, sensitivity is essentially 1.00, meaning that patients with scores below this value are unlikely to have PD under the evaluated study conditions and may be considered lower‐risk, minimizing missed cases.

At the high threshold $T_{\mathrm{high}}=0.70$, specificity approaches 0.90, supporting the use of high scores as a trigger for expedited neurological assessment or referral. Together, these ROC results suggest the potential utility of a three‐zone triage strategy (rule‐out, gray zone, rule‐in) that is intuitive for clinicians and appears stable across the evaluated cohorts; however, the clinical validity of these thresholds still requires prospective confirmation in real‐world settings. Panels b and d complement this view with PR curves, which are more informative in settings where PD prevalence is relatively low, such as primary care or community screening. The full‐cohort AUPRC values around 0.91, together with similarly high AUPRCs for the bootstrap curves, show that the model maintains a high proportion of true PD patients among those it flags as positive, even under class imbalance and reduced sample size.

The numeric summaries further emphasize the clinical impact of the chosen thresholds: at $T_{\mathrm{high}}=0.70$, the positive predictive value (PPV) is high, meaning that most patients in the rule‐in zone genuinely exhibit PD‐like handwriting and merit specialist review; at $T_{\mathrm{low}}=0.30$, the negative predictive value (NPV) is close to 1.00, so a “negative” screen is strongly reassuring. Even so, real‐world clinical applicability has not yet been established prospectively and will also depend on factors not fully captured here, including disease prevalence, comorbid motor disorders, calibration of predicted probabilities, and integration into clinical workflow. Taken together, the ROC and PR panels demonstrate that PD‐MGMA‐DSCNN may provide the basis for a promising decision‐support tool that could help rule out lower‐risk individuals, prioritize higher‐risk cases for further work‐up, and retain discriminative power under the benchmark and cross‐dataset conditions evaluated in this study, rather than proving immediate readiness for unsupervised clinical deployment.

Moreover, an important limitation is that the available public benchmark datasets do not provide a fully harmonized subject‐level clinical phenotyping for all PD cases. In particular, although PaHaW source publications report cohort‐level severity‐related information, and HandPD provides demographic/task‐level metadata, neither benchmark offers the complete set of symptom‐specific annotations that would allow us to quantify, within the present image‐based study, what proportion of cases had tremor‐dominant disease, micrographia, clinically manifest bradykinesia, or a documented medication ON/OFF acquisition state for each analyzed sample. Therefore, our findings should be interpreted as evidence of discrimination between PD and control handwriting patterns in established benchmark cohorts, not as proof of validated performance across medication states or symptom‐defined clinical subtypes. Future work should address this directly using prospectively collected handwriting cohorts with standardized neurological ratings and medication‐state annotation.

A further limitation relates to the choice of structured drawing tasks as the basis of the present pipeline. Spiral, meander, and wave tasks were selected because they were the most readily harmonizable across PaHaW and HandPD and because they provide standardized geometric constraints that reduce content‐related variability. This makes them attractive for image‐based modeling and cross‐subject comparison. At the same time, tracing‐like or template‐constrained tasks are not interchangeable with copying tasks or natural handwriting production. They may differentially engage visual feedback, visuomotor correction, movement pacing, and the speed–accuracy trade‐off, all of which are relevant in PD. As a result, the abnormalities detected by the present model may reflect not only impaired motor output, but also task‐specific demands related to guided shape reproduction. For this reason, the proposed framework should currently be interpreted as a structured drawing‐based PD assessment approach, and further work is needed to determine how its performance and interpretability would change under copying or free‐writing conditions. Future work may also explore lighter handwriting‐oriented architectures and transfer of compact representation‐learning strategies across related handwriting‐analysis tasks (Anari et al. 2022), as well as optimization‐driven multimodal extensions incorporating temporal or biosignal information.

### 5.3 Comparative Analysis

As shown in Table 7, the proposed PD‐MGMA‐DSCNN method is competitive with a diverse range of existing handwriting‐based PD detectors across traditional CNN and recurrent models and more recent attention‐ and 3D‐based designs. Early 2D CNN–SVM and hybrid deep‐learning methods by Díaz et al. (2021, 2019) report 75–87% accuracy on PaHaW, while multiple fine‐tuned 2D CNNs and a continuous convolution network achieve higher accuracy of 85%–89% (Gazda et al. 2022; Z. Li et al. 2022).

**TABLE 7:** Comparative classification performance of handwriting‐based Parkinson's disease detection methods on PaHaW, HandPD, and related datasets.

Reference	Architecture	Dataset	Metric (accuracy, %)
Díaz et al. (2021)	2D CNN and SVM	PaHaW	75.00
Kamran et al. (2021)	AlexNet fine‐tuned	HandPD and NewHandPD	99.22
Gazda et al. (2022)	Multiple fine‐tuned 2D CNNs	PaHaW	85.70
Z. Li et al. (2022)	Continuous convolution network	PaHaW	89.30
Díaz et al. (2019)	Deep learning + classical classifiers	PaHaW	86.67
X. Wang et al. (2024)	3D CNN architecture	PaHaW	86.67
Pereira et al. (2018)	CNN	HandPD	93.50
Ribeiro et al. (2019)	Recurrent neural network (RNN)	HandPD	92.20
Afonso et al. (2020)	Deep optimum‐path forest	HandPD	83.79
Saleh et al. (2024)	CNN–KNN ensemble voting classifier	Spiral–Wave	96.67
Hadadi and Poorzaker Arabani (2024)	CNN + Harris Hawks Optimization (HHO)	HandPD	91.00
Jiang et al. (2025)	Attention‐based CNN for handwriting images	Clinical handwriting images	96.50
Saadh et al. (2025)	CNN feature extractors + feature selection + ML classifiers	Spiral drawings	98.00
Chen et al. (2025)	1D CNN on smart‐pen handwriting signals	Smart‐pen handwriting signals	96.22
Our study	Multiscale gated multi‐head attention depthwise separable CNN	PaHaW	**98.23**
		HandPD	**99.42**

More recently, X. Wang et al. (2024) investigated a 3D CNN architecture to better leverage the temporal dimension of digitized handwriting trajectories, but reported PaHaW accuracy of 86.67%. Against this background, our multiscale gated multi‐head attention depthwise separable network method achieves 98.23% accuracy on PaHaW with a single unified model (Table 7), closing the remaining gap in error on this difficult clinical dataset without relying on ensemble schemes or multiple task‐specific networks. This improvement is observed while using a lightweight depthwise backbone and carefully tuned hyperparameters, rather than very deep or parameter‐heavy models.

On HandPD and related handwriting datasets, previous work also demonstrates strong but often task‐ or architecture‐specific performance. Classical CNN and RNN methods by Pereira et al. (2018), Ribeiro et al. (2019), and Afonso et al. (2020) report 83%–94% accuracy on HandPD, while optimization‐enhanced or ensemble variants such as the CNN–KNN voting classifier of Saleh et al. (2024) and the CNN combined with Harris Hawks Optimization obtain 91%–97% accuracy on spiral drawing and HandPD tasks (Saleh et al. 2024; Hadadi and Poorzaker Arabani 2024). Kamran et al. (2021) report 99.22% accuracy with a fine‐tuned AlexNet on HandPD and NewHandPD, and more recent attention‐based CNNs and multimodal feature‐selection pipelines achieve 96%–98% accuracy on clinical handwriting images or spiral drawings (Jiang et al. 2025; Saadh et al. 2025). Outside of image‐based pipelines, Chen et al. (2025) report 96.22% accuracy with a 1D CNN on smart‐pen handwriting signals, but this work requires more specialized hardware and data formats. In this context, our method achieves 99.42% accuracy on HandPD with only standard image representations and a single model configuration, matching or outperforming the state of the art while remaining compatible with tablet or scanner‐based acquisition.

Crucially, these comparisons should be taken with a grain of salt because studies vary in cohort size, inclusion criteria, task protocols (spiral, wave, meander, or mixed), and evaluation schemes (subject‐level vs. sample‐level splits, cross‐validation vs. hold‐out). Nonetheless, as can be seen in Table 7, our PD‐MGMA‐DSCNN is able to achieve consistently state‐of‐the‐art accuracy on both PaHaW and HandPD with a shared architecture while many prior methods are reported on a single dataset and a specific task (Díaz et al. 2021; Kamran et al. 2021; Gazda et al. 2022; Z. Li et al. 2022; Díaz et al. 2019; X. Wang et al. 2024; Pereira et al. 2018; Ribeiro et al. 2019; Afonso et al. 2020; Saleh et al. 2024; Hadadi and Poorzaker Arabani 2024; Jiang et al. 2025; Saadh et al. 2025; Chen et al. 2025). Furthermore, the majority of competing models do not directly optimize for cross‐dataset generalizability, principled hyperparameter selection, or model interpretability. In contrast, we couple multiscale gated attention with PD‐BayGA hyperparameter optimization and SHAP‐based explanation maps, and comprehensively evaluate our performance under multiple training protocols and bootstrap resampling. Therefore, in addition to the raw accuracy improvements reported in Table 7, the proposed approach represents a more robust and clinically grounded solution that is better equipped for deployment across different centers and handwriting acquisition conditions.

## 6 Conclusion

PD‐MGMA‐DSCNN provides a compact and interpretable framework for handwriting‐based PD detection. Using standardized image representations of spiral, meander, and wave tasks from PaHaW and HandPD, the proposed model captures both fine‐grained stroke irregularities and broader shape distortions while remaining suitable for deployment‐oriented settings. Combined with PD‐BayGA hyperparameter optimization, it achieved 98.23% accuracy on PaHaW and 99.42% on HandPD, matching or outperforming a range of recent CNN‐, RNN‐, attention‐, and ensemble‐based approaches. The SHAP analysis further suggested that the model relies on clinically meaningful handwriting abnormalities rather than obvious background cues, while the ROC and PR analyses showed the potential usefulness of the framework under clinically motivated triage thresholds.

At the same time, these results should be interpreted with appropriate caution. Despite the internal–external and bootstrap analyses, further validation is still needed in larger, prospective, multicenter cohorts with broader demographic variability and more heterogeneous acquisition conditions. Future work should also address calibration, fairness, and clinical workflow integration, and may benefit from incorporating richer temporal or multimodal information. Overall, PD‐MGMA‐DSCNN should be viewed not as a definitive deployment‐ready solution, but as a strong and explainable baseline for future digital neurology tools aimed at more reliable and transparent PD detection.

## Author Contributions

**Khosro Rezaee**: conceptualization, methodology, investigation, validation, formal analysis, visualization, writing – review and editing, writing – original draft. **Ali Khalili Fakhrabadi**: software, investigation, formal analysis, writing – original draft.

## Funding

The authors have nothing to report.

## Ethics Statement

The authors have nothing to report.

## Consent

The authors have nothing to report.

## Conflicts of Interest

The authors declare no conflicts of interest.

## Data Availability

The PaHaW and HandPD datasets are available from their original sources as described in the cited publications. Processed data and analysis scripts are available from the corresponding author on reasonable request.

## 1 Detailed Architecture of PD‐MGMA‐DSCNN

The shallow stages S1 and S2 are composed only of depthwise separable convolutions (Sep_2DConv) and max‐pooling, expanding the channel dimension from $C_{0}=3$–128 while reducing the spatial resolution from 256×256 to 64×64. A typical depthwise separable convolution layer in stage ℓ can be written as

(A1)
$$U^{\left(\ell \right)}=\sigma \left(\mathrm{BN}\left(\mathrm{PWConv}\left(\mathrm{DWConv}\left(X^{\left(\ell -1\right)}\right)\right)\right)\right)$$

where DWConv(·) applies a spatial convolution independently to each input channel, PWConv(·) is a 1×1 pointwise convolution that mixes channels, BN denotes batch normalization, and σ(·) is the nonlinear activation.

Stages S3−S5 combine additional Sep_2DConv and a small number of standard Conv2D layers with max‐pooling to progressively increase the number of channels to 512 and shrink the spatial dimensions down to 8×8, yielding deep feature maps $X^{\left(5\right)}\in R^{8\;\times \;8\;\times \;512}$ that encode complex handwriting structures, including character‐level and global shape patterns. A lightweight normalization (NormL) and residual addition (addition layer) are then applied, refining the fused representation while stabilizing training, as reflected in the configuration of the fusion block in Table A1.

**TABLE A1:** Configuration of blocks, layers, output sizes, and number of parameters in the proposed PD‐MGMA‐DSCNN model.

Blocks	Layer name	Output configuration	Parameters
Input images	Input	(None, 256, 256, 3)	0
S1	Sep_2DConv (1)	(None, 256, 256, 64)	283
	Sep_2DConv (2)	(None, 256, 256, 64)	4736
	MaxPooling2D (1)	(None, 128, 128, 64)	0
S2	Sep_2DConv (3)	(None, 128, 128, 128)	8896
	Sep_2DConv (4)	(None, 128, 128, 128)	17,664
	MaxPooling2D (2)	(None, 64, 64, 128)	0
S3	Sep_2DConv (5)	(None, 64, 64, 256)	34,176
	Sep_2DConv (6)	(None, 64, 64, 256)	68,096
	Conv2D (7)	(None, 64, 64, 256)	590,080
	MaxPooling2D (3)	(None, 32, 32, 256)	0
S4	Sep_2DConv (11)	(None, 32, 32, 512)	133,888
	Sep_2DConv (12)	(None, 32, 32, 512)	267,264
	Conv2D (13)	(None, 32, 32, 512)	2,359,808
	MaxPooling2D (4)	(None, 16, 16, 512)	0
S5	Sep_2DConv (16)	(None, 16, 16, 512)	133,888
	Sep_2DConv (17)	(None, 16, 16, 512)	267,264
	Conv2D (18)	(None, 16, 16, 512)	2,359,808
	MaxPooling2D (5)	(None, 8, 8, 512)	0
Atn1	Poi_Conv2D (19)	(None, 7, 7, 192)	393,408
	MultiHead AttentionA (1)	(None, 49, 32)	117,344
Atn2	Poi_Conv2D (14)	(None, 8, 8, 256)	131,328
	Poi_Conv2D (15)	(None, 7, 7, 192)	196,800
	MultiHead AttentionA (2)	(None, 49, 32)	117,344
Atn3	Poi_Conv2D (8)	(None, 16, 16, 256)	65,792
	Poi_Conv2D (9)	(None, 8, 8, 256)	65,792
	Poi_Conv2D (10)	(None, 7, 7, 192)	196,800
	MultiHead AttentionA (3)	(None, 49, 32)	117,344
Fusion	NormL (123)	(None, 7, 7, 32)	64
	Addition Layer	(None, 7, 7, 32)	0
Head	GlobalAveragePooling2D (1)	(None, 32)	0
	Dense (13)	(None, 2)	66

## 2 Formulation and Computational Efficiency of Depthwise Separable Convolutions

To maintain the expressiveness of the proposed model while staying computationally efficient for deployment on tablet‐ and pen‐based devices, the convolutional backbone of PD‐MGMA‐DSCNN is constructed largely of depthwise separable convolutions. Relative to regular 2D convolutions, this factorization dramatically decreases the number of parameters and floating‐point operations, enabling us to grow the network deeper and wider without exceeding the memory and latency budgets of common handwriting acquisition hardware.

In contrast, a depthwise separable convolution decomposes this operation into a depthwise and a pointwise step. In the depthwise step, a separate spatial kernel is applied to each input channel independently,

(A2)
$$U\left(i,j,m\right)=\sum_{u-1}^{K}\;\sum_{v-1}^{K}K_{\mathrm{dw}}\left(u,v,m\right)X\left(i+u-1,j+v-1,m\right),\;m=1,\text{…},M$$

where $K_{\mathrm{dw}}\in R^{K\;\times \;K\;\times \;M}$ contains one K×K filter per channel. This preserves channel‐wise stroke information, such as local edges, pen trajectories, and small tremor‐induced distortions in each feature map.

Depthwise separable convolutions closely approximate the representational power of standard convolutions at a fraction of the cost, making it feasible to deploy a deep handwriting‐based PD detector on resource‐constrained devices while still capturing the essential visual patterns associated with Parkinsonian handwriting.

## 3 Mathematical Details of the Multiscale Gated Multi‐Head Attention Module

For head h, queries, keys, and values are computed as

(A3)
$$Q_{b}^{\left(h\right)}=X_{b}\;W_{Q}^{\left(h\right)},\;\;K_{b}^{\left(h\right)}=X_{b}\;W_{K}^{\left(h\right)},\;\;V_{b}^{\left(h\right)}=X_{b}\;W_{V}^{\left(h\right)}$$

with $W_{Q}^{\left(h\right)},W_{K}^{\left(h\right)},W_{V}^{\left(h\right)}\in R^{d\;\times \;d_{h}}$ and $d_{h}=d/H$. The output of head h is:

(A4)
$${\mathrm{head}}_{b}^{\left(h\right)}=\;\mathrm{softmax}\left(\frac{Q_{b}^{\left(h\right)}K_{b}^{{\left(h\right)}^{⊤}}}{\sqrt{d_{h}}}\right)V_{b}^{\left(h\right)}\in R^{L\;\times \;d_{h}}$$

and the multi‐head attention output for branch b is obtained by concatenation and a final linear projection,

(A5)
$${\tilde{X}}_{b}=\mathrm{Conca}t_{h}\;\left({\mathrm{head}}_{b}^{\left(h\right)}\right)W_{O},\;\;{\tilde{Z}}_{b}=\;\mathrm{reshape}\left({\tilde{X}}_{b}\right)\in R^{7\;\times \;7\;\times \;d}$$

where $W_{O}\in R^{d\;\times \;d}$ and ${\tilde{Z}}_{b}$ is the attended feature map at scale b. This mechanism allows each branch to integrate information across distant regions of the handwriting image, for example linking stroke fragments at the beginning and end of a spiral or across successive letters in a word.

## 4 Additional Details of PD‐BayGA Optimization and Training Protocol

In the Bayesian phase, a Gaussian‐process surrogate $s_{t}\left(\lambda \right)$ of J(λ) is iteratively fitted to the evaluated configurations $D_{t}={\left\{\left(\lambda_{i},J\left(\lambda_{i}\right)\right)\right\}}_{i-1}^{t}$. At iteration t+1, a new candidate is proposed by minimizing a lower‐confidence‐bound acquisition function

(A6)
$$\lambda_{t+1}^{\mathrm{BO}}=\;\arg\min_{\lambda \in \Lambda }\left[\mu_{t}\left(\lambda \right)-\kappa \sigma_{t}\left(\lambda \right)\right]$$

where $\mu_{t}\left(\cdot \right)$ and $\sigma_{t}\left(\cdot \right)$ are the posterior mean and standard deviation of the surrogate and κ>0 balances exploration and exploitation. After $T_{b}$ Bayesian iterations, the top n configurations with lowest J(λ) are selected to form a narrowed search space $\Lambda_{\mathrm{narrow}}$. In the genetic phase, PD‐BayGA initializes a population $P^{\left(0\right)}=\left\{\lambda_{1}^{\left(0\right)},\text{…},\lambda_{P}^{\left(0\right)}\right\}$ by sampling from $\Lambda_{\mathrm{narrow}\;}$. At generation g, the fitness of each individual is defined as:

(A7)
$$\mathrm{Fitness}\;\left(\lambda_{i}^{\left(g\right)}\right)=-J\left(\lambda_{i}^{\left(g\right)}\right)$$

so that lower objective values correspond to higher fitness. Parents are selected via tournament selection proportional to fitness, and offspring are generated using arithmetic crossover and Gaussian mutation,

(A8)
$${\tilde{\lambda }}_{k}^{\left(g\right)}=\eta \lambda_{p_{1}}^{\left(g\right)}+\left(1-\eta \right)\lambda_{p_{2}}^{\left(g\right)},\;\;\lambda_{k}^{\left(g+1\right)}={\tilde{\lambda }}_{k}^{\left(g\right)}+\varepsilon$$

where η∼U(0,1) and ε∼N(0,Σ) with a diagonal covariance Σ adapted over generations. The best‐performing configuration after G generations,

(A9)
$$\lambda^{★}=\;\arg\min_{\lambda \in P^{\left(0\right)\cup \text{…}\cup P^{\left(G\right)}}}J\left(\lambda \right)$$

The best‐performing configuration is then selected as the final hyper‐parameter setting for PD‐MGMA‐DSCNN. BayGA and our PD‐BayGA variant have been shown to achieve better objective values more efficiently than grid search, random search, or standalone Bayesian optimization or genetic algorithms, particularly in high‐dimensional search spaces.
